# Gut Microbiota Metabolite Fights Against Dietary Polysorbate 80-Aggravated Radiation Enteritis

**DOI:** 10.3389/fmicb.2020.01450

**Published:** 2020-06-26

**Authors:** Yuan Li, Huiwen Xiao, Jiali Dong, Dan Luo, Haichao Wang, Shuqin Zhang, Tong Zhu, Changchun Zhu, Ming Cui, Saijun Fan

**Affiliations:** ^1^Tianjin Key Laboratory of Radiation Medicine and Molecular Nuclear Medicine, Institute of Radiation Medicine, Chinese Academy of Medical Sciences and Peking Union Medical College, Tianjin, China; ^2^Department of Emergency Medicine, North Shore University Hospital, Manhasset, NY, United States; ^3^Laboratory of Emergency Medicine, The Feinstein Institute for Medical Research, Manhasset, NY, United States

**Keywords:** emulsifier, radiation enteropathy, intestinal microbiota, gut microbiota metabolite, GPR43

## Abstract

Radiation therapy is a cornerstone of modern management methods for malignancies but is accompanied by diverse side effects. In the present study, we showed that food additives such as polysorbate 80 (P80) exacerbate irradiation-induced gastrointestinal (GI) tract toxicity. A 16S ribosomal RNA high-throughput sequencing analysis indicated that P80 consumption altered the abundance and composition of the gut microbiota, leading to severe radiation-induced GI tract injury. Mice harboring fecal microbes from P80-treated mice were highly susceptible to irradiation, and antibiotics-challenged mice also represented more sensitive to radiation following P80 treatment. Importantly, butyrate, a major metabolite of enteric microbial fermentation of dietary fibers, exhibited beneficial effects against P80 consumption-aggravated intestinal toxicity via the activation of G-protein-coupled receptors (GPCRs) and maintenance of the intestinal bacterial composition in irradiated animals. Moreover, butyrate had broad therapeutic effects on common radiation-induced injury. Collectively, our findings demonstrate that P80 are potential risk factors for cancer patients during radiotherapy and indicate that butyrate might be employed as a therapeutic option to mitigate the complications associated with radiotherapy.

## Introduction

Pelvic and abdominal cancers are common malignancies affecting gastrointestinal (GI), genitourinary and gynecologic functions (Pal et al., 2019). Due to their large sizes and ease of spread to other abdominal organs and the lymphatic system, pelvic and abdominal cancers are increasingly being routinely treated via radiotherapy (Liauw et al., 2013; Citrin, 2017). After radiotherapy, many patients with pelvic and abdominal cancers suffer from GI syndromes (GIS) (Peterson et al., 2010; Hauer-Jensen et al., 2014), such as malabsorption, bacterial enteritis and diarrhea, which adversely affect treatment and can even lead to death. These acute and chronic complications have become major challenges for radiation oncologists and medical physicists. Therefore, the identification of potential risk factors that may negatively influence the prognosis of cancer patients after radiotherapy and development of novel therapeutic approaches are urgently needed.

Emulsifiers are a class of detergent-like molecules that are commonly added to processed foods, pharmaceutical preparations and cosmetics. Commonly used dietary emulsifiers include Tween, Span, sodium stearyl lactate (SSL) and propylene glycol esters of fatty acids. For example, Tween 80 (or polysorbate-80, P80) is a widely used food additive that lends a smooth and stable consistency to cake, chocolate, ice cream and other packaged snacks. In addition, P80 is also used as a dispersal agent, solubilizer and stabilizer for insoluble medicinal preparations (e.g., etoposide and docetaxel injections) for cancer therapy (Iusuf et al., 2015; Al-Ali et al., 2018). Because of its potential toxicity and carcinogenicity, P80 has been used in limited doses (up to 1.0%) (Food Safety Commission [of Japan], 2007; Roberts et al., 2013). However, the growing consumption of P80 over the past half-century is associated with the increasing incidence of various chronic inflammatory and metabolic diseases (Chassaing et al., 2015), particularly in conjunction with alterations in the compositions and translocation of the intestinal microbiota (Roberts et al., 2010). Ironically, cancer patients would be given foods and medicines containing P80 during radiotherapy. Therefore, whether consumption of P80 adversely impairs the efficacy of radiotherapy remains poorly understood.

The GI tract is inhabited by a community of trillions of microbes that are collectively termed the gut microbiota (Gopalakrishnan et al., 2018). The gut microbiota provides crucial benefits to hosts, including the facilitation of food digestion and development of the metabolic and immune systems (Holmes et al., 2012; Wrzosek et al., 2013). Meanwhile, perturbance or translocation of enteric microbes may also result in a series of pathological processes (Clemente et al., 2012; Lichtman et al., 2016), such as chronic colitis (Gevers et al., 2014), *Clostridium difficile*-associated disease (Ghose, 2013; Peng et al., 2018) and Crohn’s disease (Mottawea et al., 2016). Disorders of the gut microbiota are also comorbid with a series of psychiatric illnesses and systemic metabolic diseases, such as anxiety (Heijtz et al., 2011), human immunodeficiency virus (HIV) infection (Sun et al., 2016) and non-alcoholic fatty liver disease (Chu et al., 2019). Indeed, substantial evidence suggests a positive association between radiotherapy and the development of colitis (Kirsch et al., 2010; Andreyev et al., 2013), as well as significant alterations of the composition of the gut microbiota after radiotherapy (Cui et al., 2016, 2017a).

In this study, we aimed to determine whether P80 consumption leads to detrimental alteration of the gut microbiota and exacerbates radiation-induced intestinal injury (RIII). Our observations demonstrated that P80 consumption altered the composition of the gut microbiota and worsened radiation-induced GI toxicity. Importantly, administration of butyrate by the oral route mitigated the harmful effects of P80 in irradiated animals. Taken together, our findings identified that P80 is a risk factor for cancer patients during radiotherapy and indicated that butyrate might be employed as a therapeutic option for protection against radiation-associated GI toxicity in preclinical settings.

## Materials and Methods

### Animals

This experiment tested groups (12 mice per group) of 6–8 weeks old male C57BL/6J mice, housed using Individual Ventilated Cage as six mice per cage to provide a study power of 80%. Experimental mice were treated with 200 ul P80 (1.0% in sterile water) administered via oral route for 7 days, sterile water was used as control. No specialist equipment was used. Cyclical bias were controlled as follows. Mice were randomly allocated to treatment groups, no more than six mice per cage to ensure the active area of each mouse was not less than 0.01 m^2^. Mice were maintained on clean sawdust substrates and fed an autoclaved (60 min wet cycle) pellet food (Cat#1022, HFK Bioscience, Beijing, China, ≤5%CHO, ≥18%PRO, ≥4%FAT/kg) and autoclaved filtered water (pH around 5) *ad libitum*. To prevent dirty bedding, the bedding material were changed every 2 days and mice in the same cohort were blended and re-separated every 2 days throughout the whole experiments to avoid the effects of coprophagy on gut microbiome. Food/water were replaced every 4 days. A co-housing protocol was used before the start of all experiments. Fresh murine feces were collected routinely in the morning the day after cage replacement and stored for about 30 days at −80°C until analysis.

### Polysorbate-80 (P80) Administration

P80 was purchased from Sigma (Spain, Cat#59924). In this study, P80 consumption was performed by oral gavage of 200 μl for 7 days (the concentration of P80, 1.0% in sterile water) to mice before exposed to abdominal irradiation and last until the end of the experiment. The same water was used as vehicle for the water-treated group (control).

### Radiation-Induced Intestinal Injury Model

A Gammacell^®^ 40 Exactor (Atomic Energy of Canada Lim, Canada) was used for all experiments. Male (approximately 20 g in body weight) mice were treated with a single dose of 12 Gy or 15 Gy γ-ray at a rate of 1.0 Gy/min (test by dose rate meter carried by the Exactor) total abdominal irradiation (TAI) using a specific steel chamber, and radiation dose was monitored by a dose rate meter. After irradiation, the mice were returned to the animal facility for daily observation and treatment as described above. Mice in all groups were monitored for survival status throughout the 30-day course of the experiment, and then sacrificed to collect tissue samples for gross structural and functional examinations.

### Hematoxylin and Eosin (HE) Staining

Following euthanasia, the small intestines and colons of mice were fixed in 4% buffered formalin overnight at room temperature and then embedded in paraffin. Tissues were sectioned at 5 μm thickness and dipped in hematoxylin and eosin (Solarbio, China) using standard protocols.

### Periodic Acid-Schiff (PAS) Staining

Small intestine tissues were fixed in Carnoy (60% dry ethanol, 30% chloroform, 10% glacial acetic acid) (Jiangtian Chemical Technology, China) solution overnight, and 5 μm paraffin sections were deparaffinized and oxidized in 1% periodic acid (Solarbio, China) for 10 min, after rinsing, tissues were incubated in Schiff’s reagent (Solarbio, China) for 10 min, washed in warm water, and counterstained with hematoxylin (Solarbio, China) for 30 s, washed and dehydrated before mounting.

### Fecal Microbiota Analysis

For this study, stool samples were freshly collected from five mice in different cages as described before (Cui et al., 2017b), and then stored at −80°C until use. DNA was extracted from the stool samples using the Power fecal^®^ DNA Isolation Kit (MoBio, Carlsbad, CA, United States). The DNA was recovered with 30 μl of buffer in the kit. The 16S ribosomal RNA (rRNA) V4 gene amplification and sequencing were done using the Illumina MiSeq technology. The library was sequenced on an Illumina HiSeq 2500 and 250 bp paired-end reads were generated. Sequence analyses were performed by Uparse software (Uparse v7.0.1001^1^). Sequences with ≥97% similarity were assigned to the same OTUs. Representative sequence for each OTU was screened for further annotation. For each representative sequence, the Silva123 Database was used based on RDP classifier (Version 2.2^2^) algorithm to annotate taxonomic information. Statistical difference of 16S rRNA high-throughput sequencing was assessed by Tukey’s HSD. The primers are listed in Supplementary Table S1.

### Butyrate Administration

For butyrate treated group mice, sodium butyrate (Tokyo Chemical Industry, Japan) was gavaged every day for 10 consecutive days after 12 Gy TAI. Fresh feces were collected before and after 7 days of P80 treatment, as well as 7 days after butyrate remedy, respectively, for downstream analysis.

### Antibiotic Cocktail (ABX) Administration

ABX consisted of ciprofloxacin (0.125 g/l), metronidazole (0.1 g/l), vancomycin (0.05 g/l), streptomycin (100 U/l) and penicillin (100 U/l). Mice were exposed to ABX in drinking water after TAI. The same water was used for the water-treated (TAI+P80) group.

### Short Chain Fatty Acids (SCFAs) Measurements

Quantification of short chain fatty acids was performed by high performance liquid chromatography (HPLC) (Thermo Fisher, United States) in supernatants of fecal samples reconstituted in PBS. In brief, 1 ml of fecal solution was acidified with 1/10 volume of H_2_SO_4_ (0.01 M), and passed through a condenser to isolate volatile compounds within a sample. Following filtration through 0.45 μm membrane, equal volume of samples was loaded onto the HPLC Chromatographic column: C18 AQ (4.6^∗^250 mm), sulfuric acid (0.01 M) was used as the mobile phase, and the level of SCFAs were determined by external standard calibration method.

### Fecal Microbiota Transplantation (FMT)

For FMT experiments, fresh transplant materials were prepared on the same day of transplantation within 4 h before gastric perfusion to prevent changes in bacterial composition (Cui et al., 2017b). Particularly, 200 mg of stools from the P80-treated mice was re-suspended in 2 ml sterile saline, vortexed for 30 s and allowed to stand for 30 min anaerobically. Eight-week-old male C57BL/6J recipient mice were orally administered daily with the supernatant for 10 days before 12 Gy TAI and maintained on sterile water throughout the experiment.

### Quantitative Real-Time Polymerase Chain Reaction (qRT-PCR)

Total RNA was separated from the tissues or cells using Trizol (Invitrogen, United States) according to the manufacturer’s protocol. cDNA was synthesized from total RNA using poly(A)- tailed total RNA and reverse transcription primer with ImPro-II Reverse Transcriptase (Promega, United States), according to the manufacturer’s protocol. The RT-PCR was performed using DreamTaq^TM^ Hot Start Green PCR Master Mix (Thermo Fisher Scientific, CA, United States) according to the manufacturer’s protocol. The qRT-PCR was performed according to the instructions of Fast Start Universal SYBR Green Master (Rox) (Roche Diagnostics GmbH, Germany). GAPDH was used as the housekeeping gene. The expression of gene was indicated with 2^–ΔΔCt^ and normalized to GAPDH. Specifically, the calculated 2^–ΔΔCt^ in control group was set to 1, the expression of gene in experimental group was valued as 2^–ΔΔCt(experimental group)^/2^–ΔΔCt(control group)^. The primers used in this study were listed in Supplementary Table S2.

### Residual P80 Measurements

Quantification of residual P80 was performed by HPLC in supernatants of fecal samples. The chromatographic analysis was carried out on a Dionex Ultimate 3000 (Dionex, United States), and the mobile phase consisted of 20 mM ammonium acetate and acetonitrile (9:1). Following filtration through 0.45 μm membrane, equal volume of samples was loaded onto the HPLC. Flow rate was 0.6 ml/min, and the column temperature was 30°C.

### Immunohistochemistry Staining (IHC)

Following euthanasia, the colons of mice were fixed in carnoy fixation overnight at room temperature and then embedded in paraffin. Tissues were sectioned at 5 μm thickness, and blocked with 5% BSA (Solarbio, China) for 1 h at room temperature and then incubated with primary antibodies overnight at 4°C and incubated with fluorescein isothiocyanate (FITC)-conjugated goat anti-rabbit IgG antibody (ZSGB Bio, China) for 1 h at room temperature, then stained by DAB Staining Kit (ZSGB Bio, China), followed by hematoxylin nuclear counterstaining. The primary antibody of rabbit anti-MUC2 (Abcam, United States) was used.

### Quantification of Fecal LCN2 and IL-10

Frozen fecal samples were re-suspended in PBS containing 0.1% Tween 20 (Jiangtian Chemical Technology, China) to a final concentration of 0.1 g/ml, then vortexed to produce a homogenous fecal suspension, followed by centrifuging for 10 min at 14,000 × *g* and 4°C. LCN2 and IL-10 levels were measured from the clear supernatant using Mouse Lipocalin (LCN2) ELISA kit (Solarbio, China) or Mouse IL-10 ELISA kit (Solarbio, China) according to the manufacturer’s protocol. Read the OD 450 nm value with a microtiter plate reader (Rayto, China). Specifically, with the OD value of absorbance as the ordinate (Y) and the concentration of the standard LCN2 (or IL-10) to be measured as the abscissa (X), the corresponding curve is made. The content of the LCN2 (or IL-10) to be measured in the sample can be converted from the standard curve to the corresponding concentration according to its OD value.

### Cell Culture

The human enterocyte HIEC-6 cell line was cultured in RPMI-1640 medium (Gibco, CA, United States) supplemented with 10% fetal bromide serum (FBS) (Gibco, CA, United States) at 37°C in a 100% humidified atmosphere of 5% CO_2_.

### Cell Transfection

For cell transfection, the cells were cultured in a 6-well plate for 24 h and then were transfected with siRNA. All transfections were performed using polyetherimide (PEI) (Sigma, Spain) according to the manufacturer’s protocol. si-GPR43 was synthesized by RiboBio (Guangzhou, China). The sequences of siRNA were listed in Supplementary Table S3.

### Statistical Analysis

Each experiment was repeated at least three times. Significance was assessed by comparing the mean values (6 standard deviation; SD) using Student’s *t*-test between each two cohort as follows: *p < 0.05, **p < 0.01, ***p < 0.001. Statistically difference of 16S rRNA high throughput sequencing was assessed by TukeyHSD and the data were analyzed controlling for cage-clustering. Kaplan–Meier analysis was performed for survival analysis, and significance between survival curves was determined by a log rank test. Results with *p* < 0.05 were considered statistically significant. The statistical tests were clarified in related legends. Kernel-density-violin plots were used to determine the sample sizes and data distribution (Basson et al., 2020a).

## Results

### P80 Consumption Shifts the Gut Microbial Profile

**FIGURE 1:**
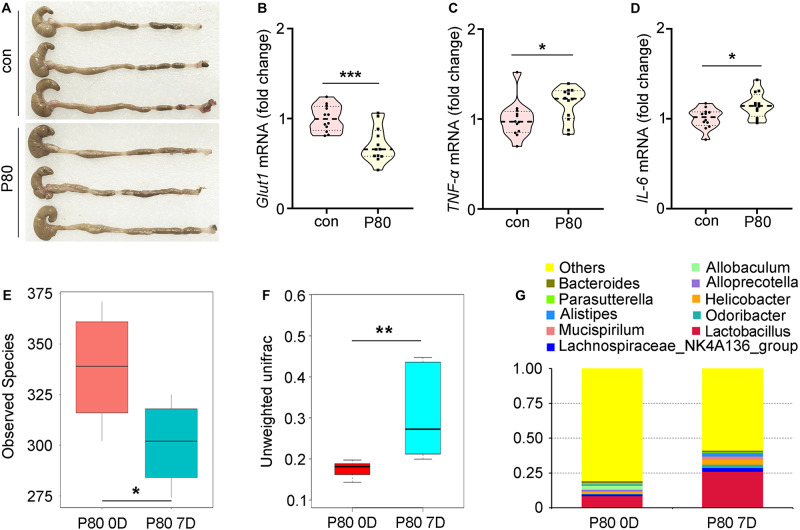
P80 consumption promotes intestinal inflammation and alters the gut microbiota. **(A)** The length of colon tissues from mice with or without consumption of P80 by oral gavage was shown. **(B–D)** The expression levels of *Glut1* **(B)**, *TNF-α* **(C)** and *IL-6* **(D)** in small intestines were examined by qRT-PCR. Significant differences are indicated: *p < 0.05, ***p < 0.001; Student’s *t*-test, *n* = 12 per group. **(E)** The observed species number of intestinal bacteria in mice before and after 7 days of consumption of P80 was examined by 16S rRNA high throughput sequencing. The top and bottom boundaries of each box indicate the 75th and 25th quartile values, respectively, and lines within each box represent the 50th quartile (median) values. Ends of whiskers mark the lowest and highest diversity values in each instance. Statistically significant differences are indicated: *p < 0.05; TukeyHSD, *n* = 5. **(F)** The β diversity of intestinal bacteria in mice before and after 7 days of consumption of P80 was examined by 16S rRNA high throughput sequencing. Statistically significant differences are indicated: **p < 0.01; TukeyHSD, *n* = 5. **(G)** The relative abundances of enteric bacteria at the phylum level in mice before and after 7 days of consumption of P80 were assessed using 16S rRNA high throughput sequencing.

In light of a recent finding that chronic P80 consumption (for 12 weeks) caused detrimental alteration of the gut microbial profile and promoted colitis (Chassaing et al., 2015), we examined whether inflammation and alteration of the gut microbiota were induced by a relatively short-term P80 challenge (for 7 days) in mice. Chronic P80-induced gains in overall weight and decrease in colon length were not observed in short-term P80-treated mice (Figure 1A and Supplementary Figure S1A; body weights not shown), while short-term consumption of P80 led to loose stools (Figure 1A). Given the important role of *Glut1* (*Slc2a1*), *Pgk*, and multidrug resistance protein (*MDR*) in the maintenance of epithelial integrity (Zaki et al., 2010; Kelly et al., 2013; Winkler et al., 2015; Cui et al., 2017b; Xiao et al., 2018), we observed a significant reduction in the expression of *Glut1* (but not *MDR* and *Pgk*) in the P80-challenged animals (Figure 1B and Supplementary Figures S1B,C). In addition, the *tumor necrosis factor-α (TNF-α)* and pro-inflammatory cytokine *interleukin 6* (*IL-6*) exhibited increased expressions in the small intestines of mice subjected to short-term P80 treatment (Figures 1C,D). Although short-term P80 consumption induced mild colitis in mice, it did not cause histopathologic epithelial damage in the intestines (Supplementary Figure S1D). Next, we performed 16S rRNA sequencing analysis to detect alterations in the intestinal microbiota after short-term P80 treatment. The results revealed a marked decrease in the numbers of intestinal bacterial species after short-term P80 consumption (Figure 1E and Supplementary Figures S1E,F). Principal component analysis (PCA) further confirmed that short-term P80 administration significantly altered the composition of the intestinal bacteria (Figure 1F and Supplementary Figure S1G), leading to increased abundances of *Lactobacillus* but decreased abundances of the *Allobaculum* genus (Figure 1G).

### P80 Consumption Aggravates RIII

**FIGURE 2:**
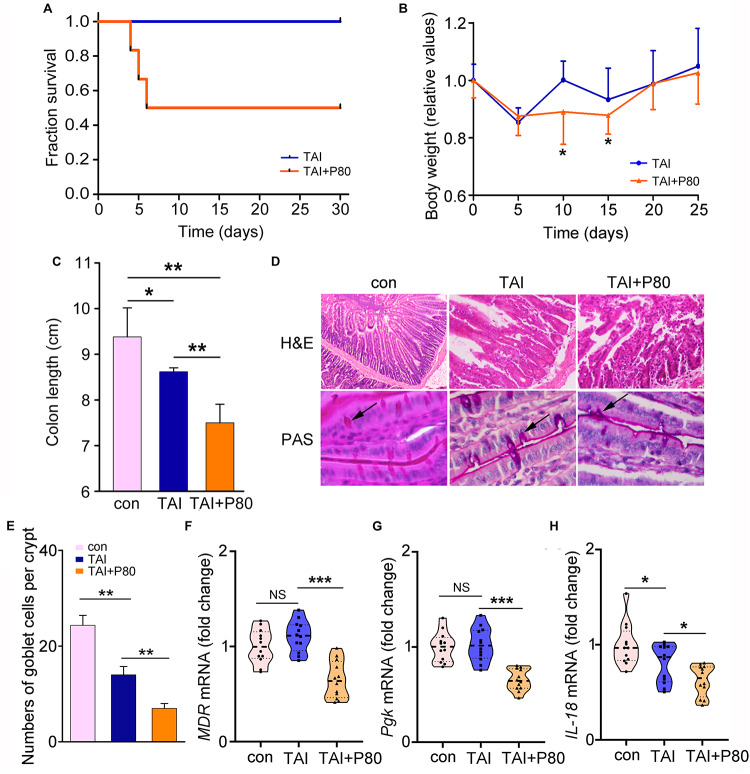
P80 consumption aggravates RIII. **(A)** Mice received 7 days of oral gavage with water or P80 before TAI. Kaplan–Meier survival analysis was performed. *p* = 0.0028 by log-rank test, *n* = 12 per group. **(B)** The body weight was measured using two-way group ANOVA (*p < 0.05), *n* = 12 per group. **(C)** The length of colon tissues from irradiated mice was measured at 30 days after TAI. Statistically significant differences are indicated: *p < 0.05, **p < 0.01; Student’s *t*-test, *n* = 12 per group. **(D)** The morphologies of the small intestines of mice were shown by HE and PAS staining. **(E)** The number of goblet cells per crypt was counted. Statistically significant differences are shown relative to the “TAI” group: **p < 0.01; Student’s *t*-test between each two cohorts, *n* = 5 per group. **(F–H)** The expression levels of *MDR* **(F)**, *Pgk* **(G)** in small intestines and *IL-18* **(H)** in colon tissues were examined by qRT-PCR. Significant differences are indicated: none significance (NS), *p < 0.05, ***p < 0.001; Student’s *t*-test, *n* = 12 per group.

To determine the effects of P80 consumption on RIII, we repetitively administered (oral gavage) P80 daily for 7 consecutive days before subjecting the mice to TAI. Notably, P80 treatment reduced the survival rate (Figure 2A, *p* = 0.0028) and impaired weight recovery (Figure 2B) in irradiated mice. At 30 days post-TAI, mice were sacrificed to assess intestinal injury. The irradiated mice with P80 challenge presented relatively short colons, indicating severe intestinal inflammation (Figure 2C and Supplementary Figure S2A). Histological analysis revealed that P80 consumption decreased the number of intact intestinal villi and goblet cells in the irradiated mice (Figures 2D,E), suggesting that oral gavage of P80 aggravated the radiation-impaired small intestinal epithelial integrity. Further determination of the levels of *Glut1*, *MDR*, and *Pgk* in the small intestines revealed significant reduction in expressions of these genes in the P80-challenged animals (Figures 2F,G and Supplementary Figure S2B). Given *IL-18* plays a role in the maintenance of epithelial integrity (Zaki et al., 2010), we observed a significant reduction in *IL-18* expression in the P80-challenged animals (Figure 2H).

### P80 Consumption Exacerbates Radiation-Induced GI Toxicity Through the Gut Microbiota

**FIGURE 3:**
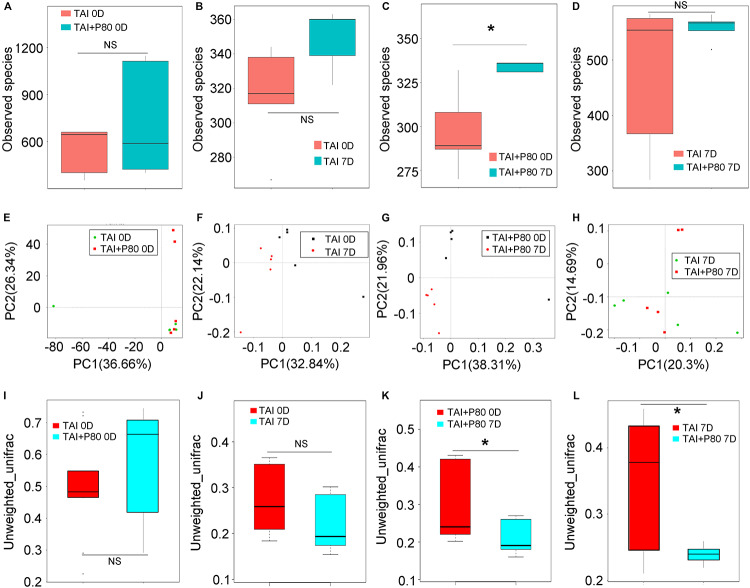
P80 alters the gut microbiota composition of irradiated mice. **(A–D)** The observed species number of intestinal bacteria was examined by 16S rRNA sequencing before (or at 7 days after) 12 Gy TAI. Mice were treated with water (TAI group) or P80 (TAI+P80 group), respectively. Statistically significant differences are indicated: none significance (NS), *p < 0.05; TukeyHSD, *n* = 5 per group. **(E–L)** The PCA **(E–H)** and β diversity **(I–L)** of intestinal bacteria in mice before (or at 7 days after) TAI were examined by 16S rRNA sequencing. The top and bottom boundaries of each box indicate the 75th and 25th quartile values, respectively, and lines within each box represent the 50th quartile (median) values. Ends of whiskers mark the lowest and highest diversity values in each instance. Statistically significant differences are indicated: none significance (NS), *p < 0.05; TukeyHSD, *n* = 5 per group.

To elucidate the mechanism underlying the P80-mediated aggravation of irradiation injury, we performed 16S rRNA sequencing using fecal pellets from the mice to characterize the composition of the gut microbiota. The mice were fed the same rodent chow and sterile water in a specific-pathogen-free (SPF) animal facility for at least 3 days to make the microbiota of the mice accordant (Figure 3A). Interestingly, TAI exposure alone did not change the α-diversity of the enteric bacteria (Figure 3B and Supplementary Figure S3A), while TAI following P80 consumption altered the α-diversity of the gut microbiota (Figure 3C and Supplementary Figure S4A). We also assessed the species number of enteric bacteria in irradiated mice with or without P80 treatment and observed no significant alterations (Figure 3D). Furthermore, PCA analysis and unweighted_unifrac algorithm further confirmed that, when the initial community configurations were similar (Figures 3E,I), TAI alone did not change the composition of the gut microbiota (Figures 3F,J), but P80 consumption significantly shifted that in irradiated animals (Figures 3G,K). Notably, although the species number of intestinal bacteria showed no changes in irradiated mice with or without P80 consumption, the PCA showed a separation of microbiota composition between the two cohorts (Figure 3H), unweighted_unifrac algorithm further confirmed that P80-challenged mice exhibited significant changes in the gut microbial composition following TAI (Figure 3L). To elaborate, TAI elevated the relative frequency of *Bacteroides* but reduced that of the *Lachnospiraceae_NK4A136* group at the genus level (Supplementary Figure S3B), whereas P80 administration increased the relative frequency of *Bacteroidetes* but decreased that of *Lactobacillus* in TAI-exposed mice (Supplementary Figure S4B). Further analysis of the gut bacterial composition at the genus level revealed increased abundances of *Candidatus_Stoquefichus*, *Parabacteroides*, *Bacteroides*, *Turicibacter*, *Erysipelatoclostridium* and *Ruminococcus_1* in the irradiated mice (Supplementary Figure S3C). In contrast, mice exposed to irradiation after P80 consumption harbored high relative abundances of bacterial genera belonging to the phyla *Firmicutes* and *Bacteroidetes*, such as *Lactobacillus*, *Anaerotruncus*, *Roseburia* and *Rikenella*, but low relative abundances of bacterial genera belonging to the phyla *Proteobacteria* and *Actinobacteria*, including *Parasutterella* and *Akkermansia* (Supplementary Figure S4C).

**FIGURE 4:**
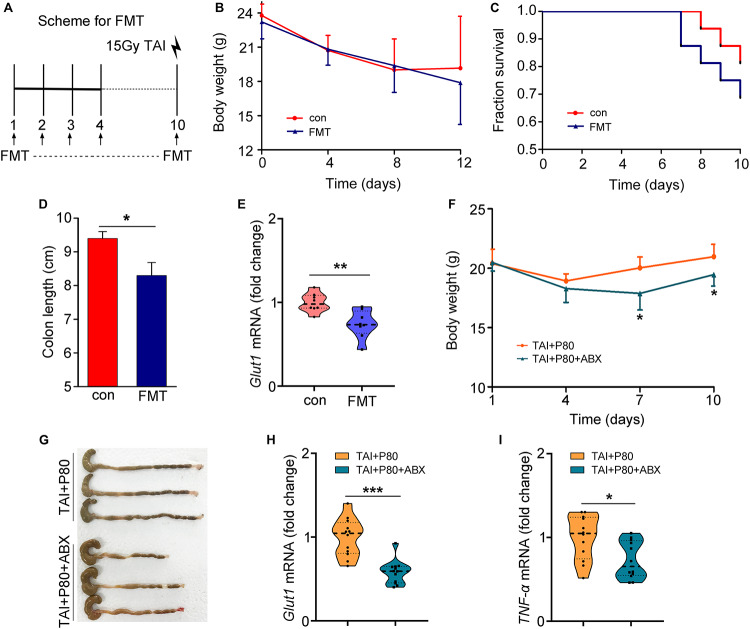
Alteration in the gut microbiota aggravates RIII. **(A–E)** Mice were separated into two groups randomly. Before 15 Gy γ ray exposure, one cohort was treated with saline as control (named saline-treated FMT), the other was treated with intestinal microbes of P80-challenged mice in saline (named P80-treated FMT). **(A)** Scheme for FMT. **(B)** Mice received 10 days of oral gavage with saline or FMT, the body weight was measured after TAI using two-way group ANOVA (*p < 0.05). *n* = 8 per group. **(C)** Kaplan–Meier survival analysis of mice was performed after TAI. *p* = 0.2589 by log-rank test between con and FMT group, *n* = 8 per group. **(D)** The length of colon tissues was measured at 15 days after TAI. Statistically significant differences are indicated: *p < 0.05; Student’s *t*-test, *n* = 8 per group. **(E)** The expression level of *Glut1* was examined in small intestine tissues by qRT-PCR. Significant differences are indicated: **p < 0.01; Student’s *t*-test, *n* = 8 per group. **(F–I)** Mice were separated into two groups randomly. Before 12 Gy γ ray exposure, mice were treated with P80 by oral gavage for 7 days, then one cohort was treated with sterile water (named TAI+P80), the other was treated with antibiotic cocktail (named TAI+P80+ABX). **(F)** Mice received water or ABX treatment, the body weight was measured using two-way group ANOVA (*p < 0.05), *n* = 12 per group. **(G)** The length of colon tissues was shown at 15 days after TAI. **(H,I)** The expression levels of *Glut1* **(H)** and *TNF-α* **(I)** in small intestine tissues were examined by qRT-PCR. Significant differences are indicated: *p < 0.05, ***p < 0.001; Student’s *t*-test, *n* = 12 per group.

To eliminate the effects of residual P80 in stools on recipients, we performed HPLC to measure the residual P80 in feces of mice that received water (control group) or P80 for 7 consecutive days. The results showed no significant difference between the two groups, indicating that P80 remains undetectable in feces after oral gavage (Supplementary Figure S5A). Thus, we performed FMT (Figure 4A) and antibiotic treatment to validate the role of gut microbes in P80-exacerbated radiation toxicity. Although FMT did not affect the body weights of the animals (Figure 4B), mice that received gut microbes from P80-challenged mice exhibited susceptibility to irradiation, leading to slightly decreased survival rates (Figure 4C, *p* = 0.2589), relatively short colons (Figure 4D and Supplementary Figure S5B) and reduced levels of intestinal integrity markers (Figure 4E and Supplementary Figures S5C,D). Consistent with the results of FMT, when we used an antibiotic cocktail (ABX, named “TAI+P80+ABX”) to eliminate the intestinal microbiota of P80-treated mice following TAI, the mice that were administered ABX exhibited increased weight loss (Figure 4F), decreased colon lengths (Figure 4G and Supplementary Figure S5E) and reduced intestinal integrity (Figure 4H and Supplementary Figures S5F,G), suggesting that alteration of the gut microbiota might be a response to P80 consumption-exacerbated GI toxicity after radiation exposure. Notably, ABX administration induced relatively low grade inflammation (*TNF-α*, *p* = 0.014; *IL-6*, *p* = 0.117) (Figure 4I and Supplementary Figure S5H); however, it might be inadequate to alleviate P80-aggravated RIII.

### Butyrate Replenishment Attenuates P80-Aggravated GI Toxicity Following Irradiation

**FIGURE 5:**
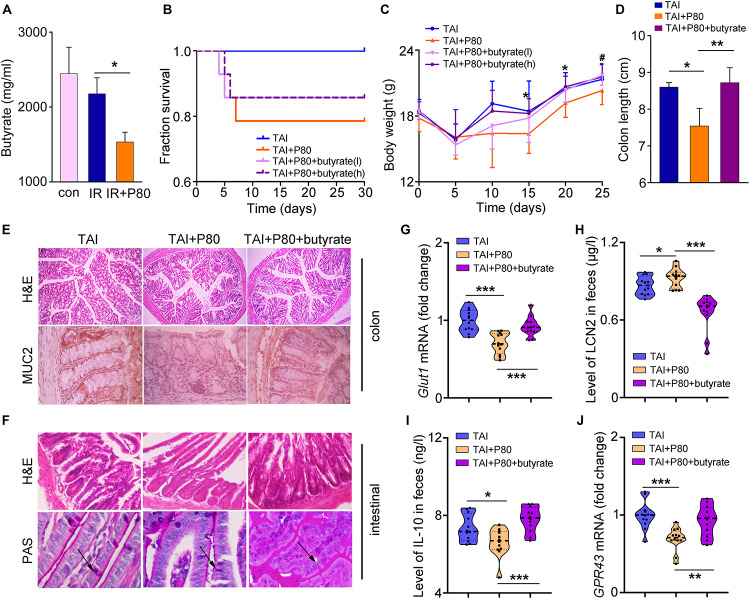
Butyrate prevents the P80-aggravated irradiation injury. **(A)** The concentration of butyrate in different fecal samples was analyzed by HPLC. Significant differences are indicated: *p < 0.05; Student’s *t*-test, *n* = 12 per group. **(B)** Kaplan–Meier survival analysis of mice after irradiation was performed. *p* = 0.0102 between TAI and TAI+P80 group by log-rank test; *n* = 14 per group. TAI group was treated with water before and after TAI as control, TAI+P80 group was treated with P80 for 7 days before irradiation and water after irradiation, TAI+P80+butyrate group was treated with P80 before irradiation and different concentrations of butyrate after irradiation (low, 3.75 mg/ml; high, 7.5 mg/ml). **(C)** The body weight of mice was measured after 12 Gy TAI using two-way group ANOVA (^#^*p* < 0.05 compared between TAI+P80 and TAI+P80+butyrate (l), *p < 0.05 compared between TAI+P80 and TAI+P80+butyrate (h); *n* = 14 per group. **(D)** The length of colon tissues was measured at 30 days after TAI. Significant differences are indicated: *p < 0.05, **p < 0.01; Student’s *t*-test. **(E)** The morphology of the colon (upper panel) was shown by HE staining. Immunohistochemical showed expression of MUC2 in the colons of mice (lower panel). **(F)** The morphology of the small intestines was shown by HE and PAS staining. The arrows pointed to the goblet cells. **(G)** The expression level of *Glut1* in small intestine tissues was examined by qRT-PCR. Significant differences are indicated: ***p < 0.001; Student’s *t*-test, *n* = 12 per group. **(H)** Fecal level of the inflammatory marker LCN2 was examined by ELISA. Statistically significant differences are indicated: *p < 0.05, ***p < 0.001; Student’s *t*-test, *n* = 12 per group. **(I)** The concentration of IL-10 in feces was examined by ELISA. Significant differences are indicated: *p < 0.05, ***p < 0.001; Student’s *t*-test, *n* = 12 per group. **(J)** The expression level of *GPR43* in the colons was examined by qRT-PCR. Significant differences are indicated: **p < 0.01, ***p < 0.001; Student’s *t*-test, *n* = 12 per group.

Given the protective effects of SCFAs against intestinal epithelial injury (Fukuda et al., 2011; Smith et al., 2013; Kelly et al., 2015), we measured the concentrations of SCFAs in fecal pellets of mice in different cohorts using HPLC. The acetate levels remained unchanged in irradiated and P80-treated animals (Supplementary Figure S6A). However, the butyrate levels decreased in the feces of irradiated animals and further decreased in the animals subjected to P80 exposure combined with irradiation (Figure 5A). In contrast, the propionate levels increased in the feces of irradiated mice and further elevated by irradiation following P80 exposure (Supplementary Figure S6B). Thus, we evaluated the therapeutic potency of butyrate toward P80-exacerbated GI injury following TAI. Using a mouse model of TAI, we observed that butyrate improved survival rate reduction (slightly, from 78.6% to 85.7%) and body weight loss of P80-challenged mice following radiation in a dose-dependent manner (Figures 5B,C), suggesting that butyrate treatment might alleviate P80-aggravated irradiation injury. We chose the effective concentration of butyrate (high, 7.5 mg/ml) to further study its role in protecting radiation enteritis. As shown in Figure 5 and Supplementary Figure S6, oral gavage of butyrate restored the length and structure of the colon (Figures 5D,E and Supplementary Figure S6C), as well as increased the number of intact intestinal villi and goblet cells in the small intestines (Figure 5F). The genes involved in maintenance of epithelial integrity were upregulated in the butyrate-treated cohort (Figure 5G and Supplementary Figures S6D–F). Moreover, butyrate treatment eliminated the alterations in LCN2 and IL-10 levels in feces driven by P80 consumption (Figures 5H,I), indicating that butyrate might be able to combat low-level intestinal inflammation.

Given that butyrate is an important energy substrate (Buzzetti et al., 2016), we observed that butyrate treatment upregulated the expression of *Mgam*, *Glut2* and *Sglt1* expression (Supplementary Figures S6G–I), which are essential genes for digestion and energy absorption (Sainz et al., 2015; Barron et al., 2016; Buzzetti et al., 2016). Previous studies have shown that the mechanism underlying the SCFAs effect partially involves G-protein-coupled receptors (GPCRs) (Macia et al., 2015). Therefore, we measured the expression of *GPR41* and *GPR43*, both of which are major GPCRs activated by SCFAs. The results showed that the levels of these two GPCRs were increased in the butyrate-treated cohort (Figure 5J and Supplementary Figure S6J).

**FIGURE 6:**
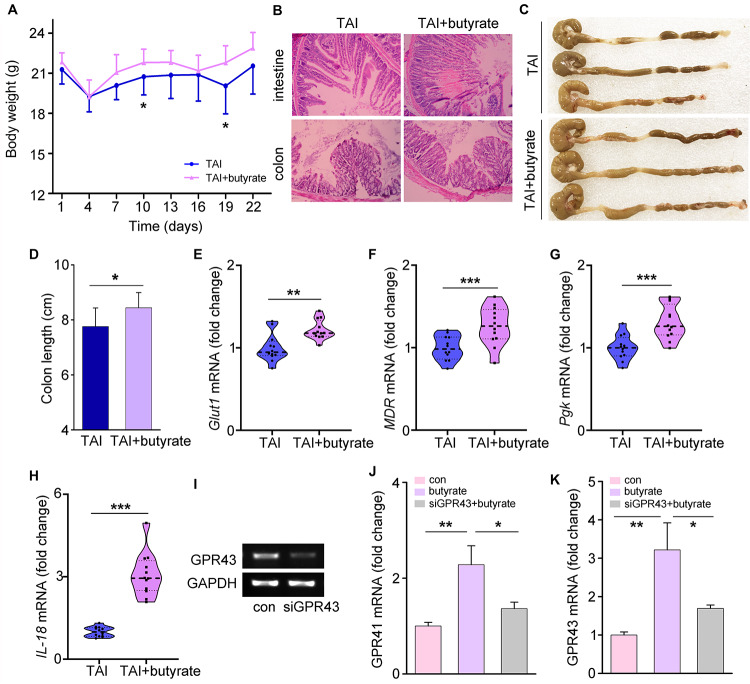
Butyrate educates P80-aggravated GI injury involving GPCRs. **(A)** The body weight of mice was measured after 12 Gy TAI using two-way group ANOVA (*p < 0.05), *n* = 12 per group. **(B)** The morphologies of the intestine (upper panel) and colon (lower panel) of mice were shown by HE staining. **(C,D)** The length of colon tissues of mice was measured at 30 days after 12 Gy TAI. Significant differences are indicated: *p < 0.05; Student’s *t*-test, *n* = 12 per group. **(E–H)** The expression levels of *Glut1* **(E)**, *MDR* **(F)** and *Pgk* **(G)** in small intestines and *IL-18* **(H)** in colon tissues of mice were examined by qRT-PCR. Significant differences are indicated: **p < 0.01, ***p < 0.001; Student’s *t*-test, *n* = 12 per group. **(I)** The expression level of GPR43 in the HIEC-6 cells with butyrate and with or without knockdown of the GPR43 expression was examined by quantitative PCR. **(J,K)** The expression levels of GPR41 **(J)** and GPR43 **(K)** in HIEC-6 cells with or without siGPR43 transfection were examined by qRT-PCR. Significant differences are indicated: *p < 0.05, **p < 0.01; Student’s *t*-test.

To determine whether butyrate could alleviate common radiation-induced injury, we performed water (as a control) or butyrate administration by the oral route. As expected, butyrate treatment resulted in significant gains in body weight and amelioration of tissue damage (Figures 6A,B). Butyrate also reduced multiple parameters of inflammation in mice, including colon length (Figures 6C,D) and expression levels of integrity markers in intestines (Figures 6E–H). To determine the mechanism underlying the modulation of radiation injury by butyrate, we supplied HIEC-6 cells with butyrate and with or without knockdown of the GPR43 expression by siRNA (Figure 6I). Similar to the results obtained in mouse models, butyrate treatment significantly upregulated the expression of GPR41 and GPR43, while GPR43 siRNA transfection downregulated GPR41 and GPR43 (Figures 6J,K).

### Oral Gavage of Butyrate Prevents P80-Mediated Alteration of the Gut Microbiota

**FIGURE 7:**
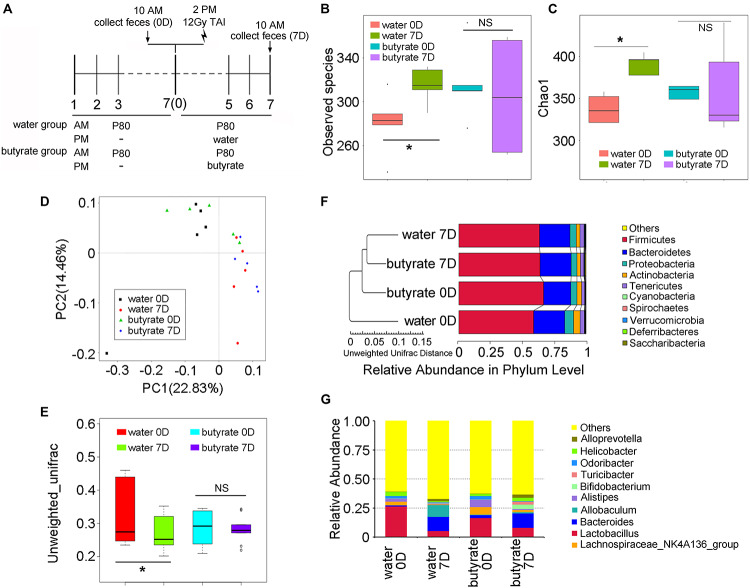
Butyrate alters the fecal microbiota. **(A)** Experimental design. “Water” group is short for “TAI+P80+water” group, “butyrate” group is short for “TAI+P80+butyrate” group. **(B,C)** The observed species number **(B)** and Chao 1 index **(C)** of intestinal bacteria of mice were examined by 16S rRNA sequencing. Statistically significant differences are indicated: non significance (NS), *p < 0.05; TukeyHSD, *n* = 5 per group. **(D,E)** The PCA **(D)** and β diversity **(E)** of intestinal bacteria in irradiated mice were examined by 16S rRNA sequencing. Statistically significant differences are indicated: non significance (NS), *p < 0.05; TukeyHSD, *n* = 5 per group. **(F)** The relative abundance of intestinal bacteria at the phylum level of irradiated mice was assessed using 16S rRNA sequencing. **(G)** The relative abundance of enteric bacteria at the genus level of irradiated mice was assessed using 16S rRNA sequencing.

To elucidate the mechanism underlying butyrate-mediated protection, we performed 16S rRNA sequencing to assess the intestinal bacterial composition of mice with or without butyrate replenishment (Figure 7A). For comparison, the gut bacterial taxonomic proportions of mice from the control group (non-irradiated and non-P80-treated) exhibited no statistically significant difference before and after 14 days of feeding (Supplementary Figures S7A–D). However, supplementation with butyrate significantly attenuated the P80-induced shift in the gut microbial profile (Figures 7B,C). PCA further confirmed that supplementation with butyrate attenuated the P80-induced intestinal bacterial composition (Figures 7D,E). Specifically, the P80-treated mice harbored increased abundances of *Proteobacteria* and decreased abundances of *Firmicutes* after TAI. However, oral gavage of butyrate stabilized the abundance of *Proteobacteria* and increased the abundance of *Firmicutes* (Figure 7F). Moreover, butyrate administration elevated the relative abundances of *Parabacteroides*, *[Eubacterium], Rikenellaceae, Turicibacter* and *Bifidobacterium* at the genus level and concurrently reduced the abundances of *Odoribacter, Anaerotruncus* and the *Lachnospiraceae_NK4A136 group* (Figure 7G and Supplementary Figure S7E).

## Discussion

During the past half-century, we have witnessed a steep rise in the consumption of food and medicine additives. These agents include spices, sweeteners, preservatives and emulsifiers, which are added to preserve the flavor or enhance the taste, appearance, or other qualities of foods and medicines. The primary basis for approving the usage of the additives is the notion that they fail to favor toxicity at concentrations reasonably greater than the approved concentrations. Of late, mounting evidence corroborates that many of these additives may have harmful effects on human health. For instance, artificial sweeteners have recently been reported to cause glucose intolerance in humans (Suez et al., 2014). Similarly, there is an on-going debate regarding a possible association between the addition of thiomersal in vaccines and the development of autism (Siva, 2012). Dietary emulsifiers, such as carboxymethylcellulose (CMC) and P80, have been reported to impair the mucus layer and alter the composition of the intestinal microbiota, resulting in colitis and metabolic syndrome (Chassaing et al., 2015). The intestinal tract is home to a complex and diverse microbial flora, which interacts with the gut epithelium to maintain an effective mucosal barrier that is important for the maintenance of homeostasis (Brown et al., 2015; Zanvit et al., 2015). Recent investigations have demonstrated that non-nutritive sweeteners and dietary emulsifiers consumption shape the gut microbiome, resulting in intestinal disturbance and inflammation, spurring the development of the metabolic syndrome and detrimentally influence anxiety-related and social behaviors (Suez et al., 2014; Chassaing et al., 2015; Holder et al., 2019). For more than 10 years, microbial configuration has been associated with radiosensibility in both humans and mouse models (Sokol and Adolph, 2018). Notably, the data presented in our previous studies have shown that regular work-rest cycle and FMT fought against radiation toxicity via modulating gut microbiome (Cui et al., 2016, 2017b). Specifically, it has been reported in our previously studies that TAI had no effect on the abundance of the intestinal bacterial of mice after 3 days (Cui et al., 2017a), 5 days (Xiao et al., 2018) and 6 or 12 days (Li et al., 2020) of TAI. In addition, recently study reported that after 1 month of TAI, there was no significant change in the abundance of gut microbiota (Lu et al., 2019). All the studies prove that TAI unchanges the α-diversity of gut microbiota in hosts. However, in light of PCA analysis in this study and principal coordinates analysis (PCoA) analysis in our another study (Li et al., 2020), TAI exposure spurred the separation of gut microbiota, suggesting that TAI might alter the composition of intestinal microbe configurations. Moreover, germ-free mice, which lack colonizing microbes, are resistant to radiation-induced lesions, indicating that the gut microbiota indeed relates to radiation-induced intestinal damage (Crawford and Gordon, 2005). Accordingly, we conjectured that P80 might be a potential risk factor for cancer patients receiving radiotherapy. To test this hypothesis, we performed TAI exposure mouse models with P80 consumption. As expected, the experimental mice exhibited more deteriorate symptom. It suggests that P80 might be an adverse agent for cancer patients receiving radiotherapy. Therefore, the medication and diet of cancer patients with radiation therapy need monitoring. In particular, the intake of P80 should be strictly limited so as not to impair the prognosis. Cage-clustering effect is a key interference factor for studies on gut microbes using mouse models (Rodriguez-Palacios et al., 2018; Basson et al., 2020b). Here, it should be noted that although we controlled the cyclical bias by maintaining relative clean bedding material and providing sterile diet and water, the cage-clustering effect always exists in this system as the limitation of studies using mouse data, unless mice are housed individually, or statistical methods are used to control for cage effects.

The human GI tract harbors trillions of microbes, constituting a bioreactor fueled by dietary macronutrients to produce bioactive molecules. Many microbial products might emerge as messengers between gut microbes and hosts, and play pivotal roles in governing the physiological processes of hosts, ranging from metabolism to brain function. For example, a great majority of 5-hydroxytryptamine is synthesized in the GI tract, modulating various biological processes, including the function of the GI, cardiac and respiratory systems as well as platelet aggregation (Kishi et al., 2013). Likewise, other microbial products, such as betaine, trimethylamine *N*-oxide (TMAO) and choline, are also involved in the development of cardiovascular diseases (Wang et al., 2011). Bile acids, which are major intestinal metabolites, may have beneficial effects on obesity and hepatic insulin sensitivity (Tagawa et al., 2015; Kusumoto et al., 2017). SCFAs are a group of major fermentation products from dietary fibers by microorganisms in the gut, serve as histone deacetylases inhibitors and GPCRs ligands governing host metabolism and immunity (Chung et al., 2016; Koh et al., 2016). In comparison with radiation-free mice here, TAI exposure downregulated butyrate levels, a member of SCFAs, which was further decreased in fecal pellets of mice subjected to P80 challenge. Since *Allobaculum* is thought to be a beneficial bacterium, which plays anti-inflammatory role, protects gut barrier function and regulates host metabolism and immunity by producing SCFAs in the intestine (Cox et al., 2014; Ma et al., 2020). Thus, P80-treated mice may harbor lower relative abundance of *Allobaculum* in the intestinal microbiota. Butyrate supplementation hinders the development of hepatitis and colitis (Zhang et al., 2016; Sheng et al., 2017), implying that butyrate might be harnessed to protect against radiation toxicity. To test this hypothesis, we performed a butyrate “rescue” experiment in which butyrate was artificially supplied via the oral route. We treated P80-challenged mice with butyrate at concentration of 3.75 mg/ml and 7.5 mg/ml and results showed that butyrate performed therapeutic effects in a dose-dependent manner. Then, we used the optimal dose of butyrate (7.5 mg/ml) throughout the study. As expected, butyrate replenishment significantly restored the P80-shifted gut microbial composition, and mitigated P80-aggravated GI toxicity in irradiated animals. Generally, radiation-induced GI tract injury represents as shortening of colon, losing of intestinal villi and decreasing of goblet cells. In addition, emulsifiers intake propelled epithelial damage of small intestine and shortened colons. All the evidence indicates that small intestine might be vulnerable to external stimulus and shows structural damage, and contraction in length of colon might be a marker for GI tract toxicity following radiation. Concerning the possible mechanism by which SCFAs modulate host responses, previous study suggests that human genome possesses approximately 800 GPCRs, and lately a cluster of four GPCR genes is identified in close proximity to the *CD22* gene on chromosome 19q13.1. Luminal SCFAs are sensed by GPCRs, releasing bioactive factors to impact satiety, inflammation and intestinal transit (Koh et al., 2016). In line with the previous implications, the underlying mechanism by which butyrate performs radiation protective effects partly dependents on binding and activating GPCR. In addition, 16S rRNA sequencing indicated butyrate replenishment reconstructed the enteric bacteria composition of irradiated mice, suggesting that the gut microbe pattern is the essential element to sustain butyrate-mediated radiation toxicity. Taken together, our findings suggest that butyrate, a metabolite of the gut microbiota, can be employed as a therapeutic agent to attenuate P80-aggravated GI injury following radiotherapy.

## Data Availability Statement

The data can be found in NCBI, under accession PRJNA636288 (https://www.ncbi.nlm.nih.gov/Traces/study/?acc=PRJNA636288).

## Ethics Statement

The animal study was reviewed and approved by Chinese Academy of Medical Sciences, Institute of Radiation Medicine.

## Author Contributions

YL, JD, DL, SZ, TZ, CZ, and MC contributed to the data curation. HX contributed to the formula analysis. MC and SF contributed to the funding acquisition. DL contributed to the methodology. YL, MC, and SF contributed to the project administration. MC supervised the study. YL wrote the original draft of the manuscript. YL, HX, JD, HW, and MC reviewed and edited the manuscript. All authors contributed to the article and approved the submitted version.

## Conflict of Interest

The authors declare that the research was conducted in the absence of any commercial or financial relationships that could be construed as a potential conflict of interest.

## Footnotes

1 http://drive5.com/uparse/

2 http://sourceforge.net/projects/rdp-classifier/
