# Supplementary material for: Gut Microbiota Metabolite Fights Against Dietary Polysorbate 80-Aggravated Radiation Enteritis
Source: Front Microbiol. 2020 Jun 26;11:1450. doi: 10.3389/fmicb.2020.01450 (PMC7332576; doi:10.3389/fmicb.2020.01450)
Supplement: Supplementary file 8 [file Data_Sheet_1.docx]

Supplementary Material

**Supplementary Fig.1** P80 exposure promotes intestinal inflammation and shapes the gut microbial profile. (A) The length of colon tissues of mice with or without consumption of P80 was measured. Significant differences are indicated: none significance (NS); Student’s *t*-test, n=12 per group. (B, C) The expression levels of *MDR* (B) and *Pgk* (C) in small intestines of mice were examined by qRT-PCR. Significant differences are indicated: none significance (NS); Student’s *t*-test, n=12 per group. (D) The morphologies of the small intestines of mice were shown by H&E. (E) The observed species number of intestinal bacteria in mice before and after 7 days of consumption of P80 was examined by 16s rRNA high throughput sequencing. The top and bottom boundaries of each box indicate the 75th and 25th quartile values, respectively, and lines within each box represent the 50th quartile (median) values. Ends of whiskers mark the lowest and highest diversity values in each instance. (F) The chao1 diversity index of intestinal bacteria in mice before and after 7 days of consumption of P80 was assessed by 16s rRNA sequencing. Statistically significant differences are indicated: * p<0.05; TukeyHSD, n=5. (G) The PCA of intestinal bacteria in mice before and after 7 days of consumption of P80 was examined by 16s rRNA high throughput sequencing.

**Supplementary Fig.2** P80 consumption aggravates RIII. (A) Colon tissues of irradiated mice with or without P80 consumption were measured at 30 days after 12Gy TAI. (B) The expression level of *Glut1* in small intestine tissues was examined by qRT-PCR. Significant differences are indicated: none significance (NS), ** p<0.01; Student’s *t*-test, n=12 per group.

**Supplementary Fig.3** TAI shapes intestinal microbiota induces intestinal injury. (A) The chao1 diversity index of intestinal bacteria in mice were examined by 16s rRNA high throughput sequencing before (or at 7 days after) 12Gy TAI. Mice were treated with water. Statistically significant differences are indicated: none significance (NS); TukeyHSD, n=5. (B) The relative abundances of enteric bacteria at the genus level in mice before (or at 7 days after) 12Gy TAI were assessed using 16s rRNA high throughput sequencing. n=5. (C) The alteration of intestinal bacterial patterns at the genus level in mice was assessed using 16s rRNA high throughput sequencing before (or at 7 days after) 12Gy TAI, n=5. The heatmap is colour-based on row Z-scores. The mice with the highest and lowest bacterial level are in red and blue, respectively. *Lachnospiraceae* is short for *Lachnospiraceae_UCG-001*, *[Eubacterium]* is short for *[Eubacterium]_coprostanoligenes_group*.

**Supplementary Fig.4** P80 shapes intestinal microbiota aggravating radiation-induced intestinal injury. (A) The chao1 diversity index of intestinal bacteria in mice were examined by 16s rRNA high throughput sequencing before (or at 7 days after) 12Gy TAI. Mice were treated with P80 before TAI. Statistically significant differences are indicated: * p<0.05; TukeyHSD, n=5. (B) The relative abundances of enteric bacteria at the genus level in mice were assessed before (or at 7 days after) 12Gy TAI using 16s rRNA high throughput sequencing. n=5. (C) The alteration of intestinal bacterial patterns at the genus level in mice before (or at 7 days after) 12Gy TAI was assessed using 16s rRNA sequencing, n=5. The heatmap is colour-based on row Z-scores. The mice with the highest and lowest bacterial level are in red and blue, respectively.

**Supplementary Fig.5** Alternations in the gut microbiota aggravate radiation damage. (A) The concentration of residual P80 in feces of mice that received water (control group) or P80 for 7 consecutive days were analysed by HPLC. Statistically significant differences are indicated: none significance (NS); Student’s *t*-test, n=12 per group. (B) Colon tissues of mice from P80-treated FMT and saline-treated FMT groups were shown at 15 days after TAI. (C, D) The expression levels of *MDR* (C) and *Pgk* (D) in small intestine tissues of mice were examined by qRT-PCR. Significant differences are indicated: ** p<0.01, *** p<0.001; Student’s *t*-test, n=8 per group. (E) The length of colon tissues of mice was measured at 12 days after TAI. ** p<0.01; Student’s *t*-test, n=12 per group. (F-H) The expression levels of *MDR* (F), *Pgk* (G) and *IL-6* (H) in small intestine tissues of mice were examined by qRT-PCR. Significant differences are indicated: none significance (NS); * p<0.05, *** p<0.001; Student’s *t*-test, n=12 per group.

**Supplementary Fig.6** Butyrate improves P80-aggravated RIII. (A, B) The concentrations of acetate (A) and propionate (B) in different fecal samples were analyzed by HPLC. Significant differences are indicated: * p<0.05; Student’s *t*-test, n=12 per group. (C) Colon tissues of mice were shown at 30 days after 12Gy TAI. (D, E) The expression levels of *MDR* (D) and *Pgk* (E) in small intestine tissues of mice were examined by qRT-PCR. Significant differences are indicated: ** p<0.01,*** p<0.001; Student’s *t*-test, n=12 per group. (F) The expression level of *IL-18* in colons of mice was examined by qRT-PCR. Significant differences are indicated: *** p<0.001; Student’s *t*-test, n=12 per group. (G-I) The expression levels of *Mgam* (G), *Glut2* (H) and *Sglt1* (I) in small intestines of mice were examined by qRT-PCR. Significant differences are indicated: none significance (NS), *** p<0.001; Student’s *t*-test, n=12 per group. (J) The expression level of *GPR41* in the colons of mice was examined by qRT-PCR. Significant differences are indicated: * p<0.05, ** p<0.01; Student’s *t*-test, n=12 per group.

**Supplementary Fig.7** Oral gavage of butyrate alters the fecal microbiota. (A-D) Mice were fed the same rodent chow and sterile water in a SPF animal facility. The observed species number (A), Chao 1 index (B) and β diversity (C), PCA (D) of intestinal bacteria of mice before and after 14 days of feeding were examined by 16s rRNA sequencing. Statistically significant differences are indicated: non significance (NS); TukeyHSD, n=5. (E) The alteration of intestinal bacterial patterns at the genus level of P80-pretreated irradiated mice with water or butyrate treatment was assessed using 16s rRNA sequencing, n=5. The heatmap is colour-based on row Z-scores. The mice with the highest and lowest bacterial level are in red and blue, respectively. *[Eubacterium]* is short for *[Eubacterium]_coprostanoligenes_group*, *Rikenellaceae* is short for *Rikenellaceae_RC9_gut_group*, *Lachnospiraceae* is short for *Lachnospiraceae_NK4A136_group*.

**Supplementary Tab.1** Primers used for sequencing.

| Gene | Primer sequence |
| --- | --- |
| 515F | GTGCCAGCMGCCGCGGTAA |
| 806R | GGACTACHVGGGTWTCTAAT |

**Supplementary Tab.2** Primers used for qRT-PCR.

| Gene | Forward primer sequence 5'-3' | Reverse primer sequence 5'-3' |
| --- | --- | --- |
| Glut1 (Mus)  Glut2 (Mus)  GPR41 (Homo)  GPR41 (Mus)  GPR43 (Homo)  GPR43 (Mus)  IL-6 (Mus)  IL-18 (Mus)  MDR (Mus)  Mgam (Mus)  Pgk (Mus)  Sglt1 (Mus)  TNF-α(Mus)  Gapdh (Homo)  Gapdh (Mus) | TATCCTGTTGCCCTTCTGC  TCAGAAGACAAGATCACCGGA  TTCTCAGCACCCTGAACTCCT  CTTCTTTCTTGGCAATTACTGGC  GCCTGGTGCTCTTCTTCATC  CTTGATCCTCACGGCCTACAT  CCGGAGAGGAGACTTCAC  CTCTTGCGTCAACTTCAA  TACGCCTACTATTACACCG  ATGGAGAGTGACGTTGTAAACAC  GGAAAACCTCCGCTTTCATGTA  CACCGAGGGCTGACTCATTC  TAGCCAGGAGGGAGAACAGA  GGAGCGAGATCCCTCCAAAAT  TGTTTCCTCGTCCCGTAGA | CCGACCCTCTTCTTTCATCTC  GTCATAGCCGAACTGGAAGGA  TTCTGCTCCTTCAGCTCCAT  CCGAAATGGTCAGGTTTAGCAA  AGGTGGGACACGTTGTAAGG  CCAGGGTCAGATTAAGCAGGAG  TCCACGATTTCCCAGAGA  GTCACAGCCAGTCCTCTT  CATCAAACCAGCCTATCTC  GGAAGCGATTTGATGTCTGGTA  GCCTTCTGTGGCAGATTCACA  TGATCCGTACACCAGTACCAC  TTTTCTGGAGGGAGATGTGG  GGCTGTTGTCATACTTCTCATGG  CAATCTCCACTTTGCCACTG |

**Supplementary Tab.3** Sequences used for siRNA.

| Gene | Sequence |
| --- | --- |
| GPR43 | CCG AUA ACC AGU UGG ACG Utt |
